# 

**Published:** 1898-03-19

**Authors:** 


**Notices of Births, Deaths, and Marriages** are inserted in this column at a charge of 2s. 6d. for an announcement not exceeding four lines.

### NOTICE TO CORRESPONDENTS.

All MS., Letters, Books for Review, and other matters intended for the Editor should be addressed The Editor, "The Hospital," The Hospital Building, 28 & 29, Southampton Street, Strand, W.C.

The Editor cannot undertake to return rejected MS., even when accompanied by stamped directed envelope.

**Notice to Advertisers and Subscribers.**

All Advertisements, Orders for Copies of The Hospital, and Business Communications should be addressed to **THE MANAGER, (Not to The Editor),** The Hospital, The Hospital Building, 28 & 29, Southampton Street, Strand, London, W.C.

The Cost of Subscription, post free, is as follows:—Per week, 2½d. per quarter, 2s. 8d.; per half-year, 5s. 3d.; per annum, 10s. 6d. Foreign:—Per annum, 15s. 2d.; payable, in all cases, in advance.

**NOTICE.—Vol. XXII. of "The Hospital" (April, 1897, to September, 1897), in handsome cloth binding, is now on sale at the Office of this Journal, price 7s. 6d. Cases for binding the Numbers contained in this Volume, 1s. 6d. Index to Vol. XXII., 2½d., post free. The Monthly Part for April will be ready on April 1st. Price 6d., by post 8d.**

### BOOKS RECEIVED.

SWAN SONNENSCHEIN AND CO.

"Epidemic Diphtheria." By Arthur Newsholme, M.D., M.R.C.P.

SAMPSON LOW, MARSTON, AND CO.

"A Manual of Static Electricity." By S. H. Morrell, M.D.
"Twentieth Century Practice." Edited by Thomas L. Stedman, M.D.
"The Care of the Sick." By Dr. T. Bellroth. Translated by J. Bentall Endean.

JAMES WILLING JUN. (LIMITED).

"Willing's Press Guide, 1898."

JOHN WRIGHT AND CO.

"The Medical Annual, 1898."

"The Medical Examination for Life Assurance." By F. de Havilland Hall, M.D., F.R.C.P.

ADAM AND CHARLES BLACK.

"Who's Who," 1898. Edited by G. Douglas Sladen.

LONGMANS, GREEN, AND CO.

"A Handbook of Midwifery." By W. R. Dakin, M.D., F.R.C.P.

ISBISTER AND CO.

"Peterborough Cathedral." By the Very Rev. W. Clavill Ingram D.D. Illustrated by Herbert Railton.

ARTHUR PEARSON.

"Exercise for Health." By H. H. Hulbert, B.A., M.R.C.S., and Luis J. Phelan.

LEA BROTHERS AND CO., NEW YORK AND PHILADELPHIA.

"Essentials of Obstetrics." By Charles Jewett, A.M., M.D., and Harold F. Jewett, M.D.

UNWIN BROTHERS.

"The Ventilation of the Metropolitan Railway Tunnels." By Robert Cox, M.P.

**Periodicals and Pamphlets.**—The Woman's Signal, Guy's Hospital Gazette, Leeds Hospital Magazine, Literary Digest, Medical News, Medicai Record, Critic, Humanitarian, Medical Press, London Hospital Gazette, Charity Organisation Review, Leisure Hour, Girl's Own Paper, Medical News, New York; Boy's Own Paper, Sunday at Home, Medical Bulletin, Treatment, Medical Times, Asylum News, Westminster Review, Zenana.

### The Student of Medicine.

**APPOINTMENTS.**

H. S. Basden, M.R.C.S.Eng., L.R.C.P.Lond., appointed Medical Officer for the Stanford-le-Hope District of the Orsett Union. C. Bastian, M.D.Lond., appointed Consulting Physician to University College Hospital, and Emeritus Professor of Medicine and Clinical Medicine at University College. W. B. Bennett, L.R.C.P., M.R.C.S., has been appointed Assistant Resident Medical Officer in the Brownlow Hill Workhouse, Liverpool. J. H. Bryant, M.D.Lond., appointed an Assistant Physician to Guy's Hospital. H. Chatterton, M.R.C.S.Eng., L.R.C.P.Lond., appointed Assistant Medical Superintendent at the St. Pancras Infirmary. G. B. Courtney, M.A., M.D., B.C., D.P.H.Cantab., L.R.C.P., M.R.C.S., appointed Honorary Surgeon to the Ramsgate and St. Lawrence Royal Dispensary. G. Crookshank, M.D.Lond., appointed Assistant Medical Officer to the County Asylum, Berry Wood, Northampton. W. Duncan, L.R.C.P., L.R.C.S.Edin., appointed Medical Officer for the Western District of the Luton Union. Dr. J. Forster, appointed Medical Officer for the Conisborough District of the Doncaster Union. J. F. Frew, M.B., C.M.Glas., appointed Medical Officer for the Sixth District of the Oldham Union. P. J. Freyer, B.A., (R.U.I.), M.D., M.Ch., appointed Surgeon to St. Peter's Hospital for Stone and Genito-Urinary Diseases, Henrietta Street. Dr. H. Gibbes, appointed Health Officer to the City of Detroit. J. Glaister, M.D.Glas., appointed Professor of Forensic Medicine in the University of Glasgow. J. H. Goodliff, M.D.Aberd., appointed Assistant Medical Officer County Asylum, Berry Wood, Northampton. Dr. Gordon, appointed Medical Officer for the Dalwood and Stockland Districts of the Axminster Union. Dr. Grosvenor, appointed Medical Officer of the Gloucester Union Workhouse. J. R. Hatfield, L.R.C.P., L.R.C.S.Edin., appointed Medical Officer of Health to the Chirbury Urban District Council. C. G. Higginson, M.D.Lond., M.R.C.S., L.R.C.P., appointed Assistant Resident Medical Officer to the Chorlton Union Workhouse. R. H. Hill, M.D.New York, L.S.A., appointed Assistant House Surgeon to the Derbyshire Royal Infirmary. W. T. Homan, L.M., L.Ch.Dublin, appointed Medical Officer for the Fourth District of the Rixbridge Union. W. E. L. Horner, M.B., B.S.Lond., M.R.C.S., L.R.C.P., appointed House Physician to the North Staffordshire Infirmary. R. A. Jackson, M.R.C.S.-Eng., appointed Medical Officer for the Kirby Moorside District and the Workhouse of the Kirby Moorside Union. Dr. H. C. Jarvis, appointed Medical Officer for the St. George's District of the Camberwell Union. W. J. C. Keats, M.R.C.S., L.R.C.P.-Lond., D.P.H., appointed Medical Superintendent to the Havil Street Infirmary and Gordon Road Workhouse of the Camberwell Union. Dr. H. McKay, appointed Medical Officer for the South-East District of the Sheffield Union. F. W. Martin, M.R.C.S.Eng., L.R.C.P.Ed., appointed Medical Superintendent of the Brighouse Joint Hospital for Infectious Diseases. J. Middlemass, M.A., M.B., C.M., F.R.C.P.E., Senior Assistant Physician, Royal Edinburgh Asylum, appointed Medical Superintendent of Sunderland Borough Asylum. S. G. Morris, M.D., M.S.Edin., appointed Medical Officer and Public Vaccinator for the Western District of the Llandilo Fawr Union. D. C. Muir, M.D., D.P.H., appointed Medical Officer for the Abertillery District of the Bedwellty Union. W. W. Ord, M.D.Oxon., appointed Physician to the Salisbury Infirmary. W. D. Rose, M.B., C.M.Glasg., appointed Medical Officer of the Workhouse of the Luton Union. J. S. R. Russell, M.D.Edin., F.R.C.P., appointed Assistant Physician to University College Hospital. J. M. Schaub, M.R.C.S.Eng., L.R.C.P.Lond., appointed Second Assistant Medical Officer at the Parish of St. Leonard (Shoreditch) Infirmary. T. Sidebottom, M.B., C.M., appointed Medical Officer for the No. 6 District of the Lincoln Union. Dr. A. H. Smith, appointed Medical Officer for the Yarm District of the Stokesley Union. C. N. Thomas, B.A., M.B., B.C., appointed Certifying Factory Surgeon to the Lydney District, and Surgeon to Lydney Tin Plate Works. W. Turner, M.B., B.S.Lond., appointed Surgeon to the Royal Hospital for Diseases of the Chest, City Road, E.C. S. K. Vines, L.S.A., appointed Junior House Surgeon to St. Mary's Children's Hospital, Plaistow. H. R. Wadd, M.R.C.S., L.R.C.P., appointed Anæsthetist to the Great Northern Central Hospital, Holloway Road, N. E. C. Walter, M.R.C.S.Eng., L.R.C.P.Lond., appointed Medical Officer for the Cholesey District of the Wallingford Union. J. H. Whitaker, M.B. (R.U.I.), appointed Assistant Medical Officer to the North-Western Fever Hospital, London. W. Wrangham, M.B.Lond., M.R.C.S., L.R.C.P., appointed House Physician to Leicester Infirmary.

